# Assessing the Nepalese health system’s readiness to manage gender-based violence and deliver psychosocial counselling

**DOI:** 10.1093/heapol/czae003

**Published:** 2024-01-31

**Authors:** Keshab Deuba, Rachana Shrestha, Reena Koju, Vijay Kumar Jha, Achyut Lamichhane, Devika Mehra, Anna Mia Ekström

**Affiliations:** Department of Global Public Health, Karolinska Institutet, Stockholm, Widerströmska Huset Tomtebodavägen 18 A, Plan 3, Solna 17165, Sweden; Public Health and Environment Research Centre (PERC), Sanepa-2, GPO Box 8975, EPC 450, Lalitpur, Bagmati, Nepal; Knowledge to Action (K2A), Sanepa-2, Lalitpur, Bagmati 4700, Nepal; Department of Global Public Health, Karolinska Institutet, Stockholm, Widerströmska Huset Tomtebodavägen 18 A, Plan 3, Solna 17165, Sweden; Public Health and Environment Research Centre (PERC), Sanepa-2, GPO Box 8975, EPC 450, Lalitpur, Bagmati, Nepal; Knowledge to Action (K2A), Sanepa-2, Lalitpur, Bagmati 4700, Nepal; Public Health and Environment Research Centre (PERC), Sanepa-2, GPO Box 8975, EPC 450, Lalitpur, Bagmati, Nepal; Knowledge to Action (K2A), Sanepa-2, Lalitpur, Bagmati 4700, Nepal; Health Directorate, Ministry of Social Development, Sapahi, Dhanusha, Janakpur, Madhesh Province 45600, Nepal; Public Health and Environment Research Centre (PERC), Sanepa-2, GPO Box 8975, EPC 450, Lalitpur, Bagmati, Nepal; Knowledge to Action (K2A), Sanepa-2, Lalitpur, Bagmati 4700, Nepal; MAMTA Health Institute for Mother and Child, New Delhi 110048, India; Division of Social Medicine and Global Health, Department of Clinical Sciences, Lund University, Malmö, Box 117, Lund 221 00, Sweden; Department of Global Public Health, Karolinska Institutet, Stockholm, Widerströmska Huset Tomtebodavägen 18 A, Plan 3, Solna 17165, Sweden

**Keywords:** Nepal, violence against women, intimate partner violence, health facility readiness, preparedness of health care providers, mental health, concurrent triangulation design

## Abstract

Violence against women (VAW), particularly intimate partner violence (IPV) or domestic violence, is a major public health issue, garnering more attention globally post-coronavirus disease 2019 (COVID-19) lockdown. Health providers often represent the first point of contact for IPV victims. Thus, health systems and health providers must be equipped to address survivors’ physical, sexual and mental health care needs. However, there is a notable lack of evidence regarding such readiness in Nepal. This study, utilizing a concurrent triangulation design, evaluated the readiness of public health facilities in Nepal’s Madhesh Province in managing VAW, focusing on providers’ motivation to offer psychosocial counselling to survivors. A cross-sectional study was conducted across 11 hospitals and 17 primary health care centres, where 46 health care providers were interviewed in February–April 2022. The study employed the World Health Organization’s tools for policy readiness and the Physician Readiness to Manage IPV Survey for data collection. Quantitative and qualitative data were collected via face-to-face interviews and analysed using descriptive and content analysis, respectively. Only around 28% of health facilities had trained their staff in the management of VAW. Two out of 11 hospitals had a psychiatrist, and a psychosocial counsellor was available in four hospitals and two out of 17 primary health care centres. Two-thirds of all health facilities had designated rooms for physical examinations, but only a minority had separate rooms for counselling. Though a few health facilities had guidelines for violence management, the implementation of these guidelines and the referral networks were notably weak. Hospitals with one-stop crisis management centres demonstrated readiness in VAW management. Health providers acknowledged the burden of IPV or domestic violence and expressed motivation to deliver psychosocial counselling, but many had limited knowledge. This barrier can only be resolved through appropriate training and investment in violence management skills at all tiers of the health system.

Key messages

- Although gender-based violence (GBV) and its mental health consequences have been considered a global concern, there is a dearth of evidence about the readiness of public health facilities to identify and provide assistance to women experiencing violence and its consequences in low- and middle-income countries such as Nepal.
- A framework devised for health facility readiness suggested that health facilities without one-stop crisis management centres (OCMCs) were unprepared to identify and manage violence survivors and make necessary referrals. Even health facilities with OCMCs are not fully equipped to provide optimal care to violence survivors.
- This finding also highlights that health care providers in public health facilities in Madhesh Province of Nepal were motivated to deliver psychosocial counselling to women experiencing violence; however, there was a gap in their knowledge about mental health.
- This calls for the strengthening of public health facilities along with the provision of training to health care providers for identifying and managing women experiencing violence with appropriate treatment and care along with psychosocial counselling to manage mental health consequences.

## Introduction

Women and girls globally face a higher risk of violence within their own homes from intimate partners or other family members than from strangers (National Research Council, 1996; Drakulich, 2015). Violence against women (VAW), particularly incidents of intimate partner violence (IPV) and sexual violence, constitutes a significant public health concern and is a serious infringement of fundamental human rights. The World Health Organization (WHO) has defined IPV as the behaviour within an intimate relationship (including current and former spouses and partners) that causes physical, sexual or psychological harm. This encompasses acts of physical aggression, sexual coercion, psychological abuse and controlling behaviours (World Health Organization, 2022a). Similarly, the United Nations Declaration on the Elimination of VAW (United Nations, 1994) defines gender-based violence (GBV) as any act resulting in, or is likely to result in, physical, sexual or psychological harm or suffering to women. This includes threats, coercion or arbitrary deprivation of liberty, whether occurring in public or in private life.

According to the 2021 VAW Prevalence Estimates by the WHO, ∼30% of the global female population—equating to 736 million women worldwide—have encountered instances of IPV or non-partner sexual violence (World Health Organization, United Nations and Human Reproduction Programme, 2021). VAW may have deleterious effects on the physical, psychological, sexual and reproductive well-being of women, and is a universal public health issue that profoundly impacts the lives of women and children across the globe (Sardinha *et al*., 2022).

The health sector plays a crucial role in providing or referring to medical treatment and psychological care for survivors of violence (Sikder *et al*., 2021) and serves as an entry point for referral to other support services that they may require beyond immediate medical care (World Health Organization, 2021).

VAW has increased due to COVID-19 precautions, such as lockdowns and mobility restrictions, which disproportionally affect vulnerable populations, especially women and girls (UN Women, 2021). Violence can have severe physical, mental and sexual health consequences, ranging from immediate to long-lasting injuries. Multiple studies across the world have found significant associations between IPV and ill health such as pregnancy complications including unintended pregnancies, unwanted abortion, sexually transmitted infections and chronic pelvic pain (World Health Organization, 1997; Martin-Engel *et al*., 2021; Potter *et al*., 2021). Mental health problems such as post-traumatic stress disorder, depression, anxiety, eating disorders and suicidal ideation are other major consequences of violence (Ellsberg and Emmelin, 2014; Potter *et al*., 2021). These problems can go unnoticed, unlike physical health issues. Mental disorders are the seventh leading cause of disability-adjusted life years worldwide and the second leading cause of years lived with disability (Ferrari *et al*., 2022). Additionally, women are more likely than men to suffer from mental disorders such as depression, anxiety and eating disorders (Ferrari *et al*., 2022).

Mental health constitutes a significant public health issue in Nepal, carrying a substantial burden of mental health problems (Ministry of Health and Population and Department of Health Services, 2023). A nationally representative survey of 9200 adults indicated that ∼10% had experienced some form of mental disorder during their lifetime, while around 4.3% were currently grappling with some form of mental health issues (Nepal Health Research Council, 2020). The provision of public mental health care in Nepal is overseen by the Epidemiology and Disease Control Division of Nepalese Ministry of Health and Population. Over the past decade, the government of Nepal has significantly increased its commitment to mental health services by incorporating mental health to the list of basic health services and emergency health service packages. In 2017, a government mental health policy was drafted, building on the first such policy from 1996. However, the implementation of this policy is pending, awaiting cabinet approval (Singh and Khadka, 2022). To bridge this gap and provide a strategic and clear action for addressing challenges in mental health sector, the National Mental Health Strategy and Action Plan (2020) outlines Nepal’s approach to mental health care. Specialized mental health services in the country predominantly exist in tertiary care hospitals and urban areas (Luitel *et al*., 2015). Despite intentions for mental health services to be integrated into primary health care facilities across the country, evidence indicates limited delivery of such services at primary health care centres (Luitel *et al*., 2015). According to current strategy, a range of psychotropic drugs, including antipsychotics, antidepressants, anxiolytics, mood stabilizers and antiepileptics, should be made available at all levels of health facilities; however, the distribution of these drugs is hampered by frequent stockouts and the absence of adequately skilled human resource to prescribe these medications (World Health Organization, 2022b).

Psychosocial counselling has proven effective in preventing chronic mental trauma among women who experience violence, especially IPV (Ogbe *et al*., 2020). However, there is a shortage of trained mental health service providers and other necessary resources in many low- and middle-income countries, including Nepal (Solotaroff and Pande, 2014; Uprety and Lamichhane, 2016; Singla *et al*., 2017). Although Nepal has increased the number of psychiatrists from 39 in 2008 to 200 in 2020, these numbers are insufficient to meet the high demand for mental health services (Rai *et al*., 2021), which calls for alternative interventions and lower-level health cadres. There is evidence that psychosocial counselling delivered by non-specialist mental health care workers, lay community health worker’s and master’s level social workers has been effective in preventing chronic mental trauma among women experiencing violence in other countries, such as the USA, Kenya, Tanzania, Uganda, Sri Lanka, India and Pakistan (Kiely *et al*., 2010; Bryant *et al*., 2017; Singla *et al*., 2017).

In Nepal, VAW is a prominent public health concern. According to the 2021 WHO country-specific prevalence estimates, 25–29% of women aged 15–49 years who have ever been in a partnership have reported experiencing lifetime physical or sexual, or both, IPV (Sardinha *et al*., 2022). The 2022 Nepalese Demographic and Health Survey shows that 23% of women between the ages of 15 and 49 years, or ∼1.9 million women, have experienced instances of physical violence since they were 15 (Ministry of Health and Population and New Era, 2022). Additionally, 8% of women in this age are victims of sexual violence at some point in their lives. The same survey ascertained that overall, 27% of women have endured physical, sexual or emotional violence from their current or most recent husband or intimate partner. However, the prevalence of IPV varies across regions, with the highest rates observed in the Madhesh Province at 46%, and the lowest in the Gandaki and Bagmati provinces, standing at 19% (Ministry of Health and Population and New Era, 2022).

In Nepal, the provision of mental health services is limited, and there is a stigma associated with mental health problems (Uprety and Lamichhane, 2016; Kumari Sah *et al*., 2020), making it difficult for many girls and women to disclose their experiences of violence and mental health problems to health care providers. This is compounded by patriarchal societal norms and fear of judgement, escalation of violence and the need to protect their families’ reputation (Rishal *et al*., 2016; UNDP/UNFPA/UNOPS/UN Women, 2021). The 2021 National Health Facility Survey revealed that merely a quarter of all health facilities in Nepal provide mental health services (Ministry of Health and Population, New Era and ICF, 2022). Among these, only 27% have established guidelines, and merely 16% of health workers have undergone recent training in mental health care. Furthermore, half or less of these health facilities maintain any of the essential medicines necessary for treating mental illnesses. Moreover, recent findings from the 2022 National Demographic and Health Survey underscore the severity of the situation, indicating that a mere 7% of women exhibiting symptoms of anxiety or depression reported seeking help from health care providers in Nepal (Ministry of Health and Population and New Era, 2022). In many other countries, victims of violence may seek shelter in safe houses and legal aid to punish the perpetrator, but this rarely happens in Nepal, where VAW is considered a private issue (Rishal *et al*., 2018; Dahal *et al*., 2022). Nevertheless, women experiencing violence in Nepal often seek treatment for their physical injuries at public health facilities, which are the first contact point for many of them (Joshi, 2009; Ministry of Health and Population, 2015). However, health care providers lack knowledge and skills to identify GBV as a possible cause of repeated injuries with vague explanations; also, women do not disclose their experience of violence owing to the stigma, feeling of being judged or not believed and confidentiality concerns that calls for a need of better coordination among health care providers regarding services to meet the needs of women experiencing violence (World Health Organization, 2013; 2017; Hegarty *et al*., 2020).

Different tiers of public health facilities exist in Nepal, and while there are psychiatrists and psychologists at specialized hospital at the federal and provincial levels (Department of Health Services, 2019), the same does not hold true at peripheral public health facilities. Therefore, health care providers at all levels must be trained to identify signs of violence and respond accordingly. The WHO encourages providers to have private conversations with women who have injuries or conditions consistent with violence to improve identification of GBV cases (World Health Organization, 2017). Health care providers dealing with violence must be knowledgeable about services to make proper referrals to other health facilities for specialized treatment and counselling for psychological distress management. They also need to be informed and coordinate with legal networks, social welfare systems and rehabilitative centres to protect and prevent their patients from further harm or escalation of violence (World Health Organization, 2013; 2017).

In Nepal, public hospital-based one-stop crisis management centres (OCMCs) were established under the Ministry of Health and Population in 2011 to prevent GBV and provide appropriate services to its survivors. These services include immediate medical treatment, psychosocial counselling, legal counselling, safe home, security and rehabilitation, education and empowerment (Ministry of Health and Population and Population Division, 2011). According to the 2023 annual report, there are 88 OCMCs across 77 districts in the country (Ministry of Health and Population and Department of Health Services, 2023). While the provision of mental health services is a significant component of this programme, these services are often limited to basic psychosocial counselling services (Luitel *et al*., 2015). Several challenges hinder the delivery of OCMC service at district hospital level. These include the lack of safe housing for survivors of GBV, insufficient psychiatric care for GBV survivors, delays in response from the law and justice sector, ineffective implementation of existing laws and regulations and insufficient legislative support for GBV cases (Dangal *et al*., 2021).

Existing frameworks for assessing health systems’ readiness to manage IPV are insufficient, often focusing on disease-specific (Ghimire *et al*., 2020), contextual or health service aspects. Thus, for this assessment, we adapted the WHO health system building blocks framework (World Health Organization, 2017). WHO framework was previously employed in a study on IPV in Palestine (Colombini *et al*., 2020), and make-up key references for our study’s design and the questions used to measure various dimensions within this framework were crafted by synthesizing insights from both the WHO and the Palestinian study. However, a new framework aiming to enhance the quality of care for IPV has recently emerged, following testing in Brazil and Palestinian territories (Colombini *et al*., 2022). Additionally, our assessment incorporated the Physician Readiness to Manage IPV Survey (PREMIS) (Short *et al*., 2006).

Overall, this readiness assessment aims to contribute to the development of evidence-based policies and programmes to improve the quality and accessibility of services for women experiencing violence in Nepal.

The specific objectives of this assessment were:

1. To identify the current knowledge, attitudes and practices towards IPV among health care providers in Madhesh Province as well as their confidence in dealing with and managing IPV cases.
2. To determine the availability and accessibility of services for women experiencing violence in public health facilities, including the availability of resources such as referral pathways, medical equipment and medication.
3. To assess the capacity of health facilities to deliver psychosocial counselling services for women experiencing violence, including the availability and accessibility of trained counsellors and the adequacy of the counselling services provided.
4. To identify the barriers and facilitators to the provision of GBV services in public health facilities, including policy and regulatory factors, infrastructure and resources and social and cultural factors.
5. To provide recommendations for improving the readiness of public health facilities and providers to deliver GBV services, including strategies for strengthening health systems and addressing the barriers identified to help women in coping with previous experiences of violence, developing safety plans to avoid being revictimized and improving their overall quality of life.

## Materials and methods

### Study design

This was a cross-sectional study with a concurrent triangulation-mixed method design (Creswell, 2009) employing both quantitative and qualitative approaches. Quantitative data were obtained via surveys, while qualitative data were obtained from in-depth interviews with health care providers. Additionally, health facility visits were performed to review recording and reporting forms, the availability of guidelines, information, education and communication materials pertaining to the management of violence and mental health services.

### Study setting and area

This study was conducted in all eight districts of Madhesh Province in Nepal (Saptari, Siraha, Dhanusha, Sarlahi, Rautahat, Bara, Mahottari and Parsa). Details on the population size of each district are presented in Table 1. Madhesh Province was selected because of highest rates (46%) of IPV observed in this province as compared to the other six provinces according to nationwide demographic and health survey in 2021 (Ministry of Health and Population and New Era, 2022).

**Table 1.** Name of districts along with respective population size

S.N.	District	Population size
1	Saptari	706 255
2	Siraha	739 953
3	Dhanusha	867 747
4	Mahottari	706 994
5	Sarlahi	862 470
6	Rautahat	813 573
7	Bara	763 137
8	Parsa	654 471

Source: Central Bureau of Statistics, Nepal, 2021.

We included 12 hospitals within the province and 17 primary health care centres across all districts in Madhesh. Of these 12 hospitals, one was a large federal hospital, seven were provincial hospitals and four were district level hospitals.

### Study population

Health care providers, particularly nurse and auxiliary nursing midwives (ANMs), were the major study population in this assessment. However, health care providers trained in psychological counselling who were active in the management of GBV were also included.

### Study duration

The total duration of the assessment was 3 months from February 2022 to April 2022.

#### Sampling approach

Of the total public hospitals and primary health care centres in Madhesh Province, all public hospitals except one and 17 randomly selected primary health care centres were included in the study. From the selected public health facilities, it was envisaged to interview two health care providers from each public health facility, one staff nurse and one ANM. However, we could not interview any health care provider in one hospital, as the medical superintendent was reluctant to grant permission. In addition, based on the availability of health care providers in the maternity department and OCMCs on the day of assessment, we were only able to interview 46 health care providers (staff nurses, ANMs and psychosocial counsellors) from the remaining 28 public health facilities.

### Data collection tools and techniques

A survey tool was designed based on a modified version of the PREMIS including perceived preparedness and knowledge of providers to manage IPV using 10 vs 8-item domains (Short *et al*., 2006) and WHO’s GBV/mental health management infrastructural assessment tools (World Health Organization, 2014; 2017). Similarly, the motivation of providers to offer IPV/domestic violence (DV) services and pursue further training was examined by conducting in-depth interviews with two providers from each selected facility. These interviews followed a semi-structured guide that centred on health care provider’s motivation in managing IPV/DV and its associated consequences.

All interviews were conducted in Nepali, face-to-face, by the study team at the respective health facilities where the providers worked. Following written consent, the in-depth interviews were audio-recorded and typically lasted between 25 and 30 min.

### Data management and analysis

The quantitative data were collected on paper, entered into EpiData software, double-checked, extracted into Excel and analysed descriptively using SPSS version 16. Socio-demographic data have been presented on the basis of frequency, percentage, mean and standard deviation, median and interquartile range. Health facility readiness-related questions were assessed on the basis of the framework devised for the assessment that is given in Table 2. The results were then presented in descriptive form. Similarly, the qualitative data were transcribed, translated into English and analysed using content analysis (Prasad, 2008). Open coding was conducted independently by two researchers (R.K. and R.S.). The identified codes were subsequently deliberated within the assessment team to reach consensus and organized into themes aligned with the study objectives. Emerging themes were also incorporated. These thematic findings were then amalgamated with the descriptive analysis to comprehensively evaluate the readiness of health care facilities and providers, utilizing both the readiness framework developed for the study and the PREMIS questionnaire. The analysis was carried out collectively by members of the study team (K.D., R.K., R.S. and A.L.).

**Table 2.** Health system readiness to offer appropriate services to women experiencing GBV (IPV/DV) and mental health problems in Nepal

Health system building blocks	Readiness questions	Sources of data used for each building block
Service delivery	– Does the facility have a OCMC? – Are there any services available for the management of IPV/DV in the public health facility?	Survey questionnaire administration to health care providers, interaction with public health facility in charge
Health workforce	– Are there any trained health care providers for IPV/DV and GBV management? – Is there a trained health care provider in the public health facility who can provide psychosocial support to women experiencing IPV/DV? – Is there a psychiatrist, psychologist or psychological counsellor in the public health facility? – Are health care providers motivated and prepared to address IPV/DV within their work?	Survey questionnaire and in-depth interviews with health care providers
Information	– Is there a mechanism for recording and reporting identified cases of IPV/DV?	Survey questionnaire administration to health care providers, review of recording and reporting forms
Medical products, vaccines and technologies	– Are drugs for management of mental health problems available in public health facilities? – Are vaccines for tetanus prophylaxis and hepatitis B available in public health facilities? – Are lab facilities for different sexually transmitted infections available? – Are there separate rooms for physical examination, genito-anal examination and counselling?	Survey questionnaire administration to health care providers
Leadership and governance	– Does the facility have IPV/DV management guidelines? – Are health care providers interested in delivering psychosocial counselling to women experiencing IPV/DV?	Survey questionnaire and in-depth interviews with health care providers
Coordination	– Is there coordination among higher level health facilities for appropriate referral? – Is proper collaboration in place with other legal organizations, police and safe shelter homes?	Survey questionnaire administration to health care providers

#### Health system readiness framework

A framework to assess health system readiness and provider motivation to provide counselling was developed based on the WHO’s manual for health workers, ‘Strengthening health systems to respond to women subjected to IPV or sexual violence’ (World Health Organization, 2017). This framework earlier adopted by a readiness assessment in the Palestine (Colombini *et al*., 2020) helped design the questions for our assessment. The adapted framework includes the WHO’s health system building blocks plus a component of coordination (Table 2).

### Ethical considerations

Prior to conducting the interviews, written informed consent was obtained from the health care providers. They were informed that they had the option to withdraw from the study at any time. Their confidentiality and privacy were also maintained throughout the data management and analysis processes. Additionally, the audio recordings of the interviews were securely kept in password-protected devices.

## Results

The results are presented in two main sections. Firstly, socio-demographic findings are outlined, followed by the readiness assessment results based on the adapted readiness framework for this study.

### Socio-demographic findings

The majority of the health care providers interviewed were ANMs (52%), and others were nurses (44%). About half of them had a proficiency certificate and their mean age was 32 years with standard deviation of 6.43. The median number of patients attended by the providers was 70 (interquartile range = 68.75), but 37% of them examined ≥100 patients in a week. Most had worked at their current health facility for between 1 and 5 years (Table 3).

**Table 3.** Background characteristics of the health care providers (*N* = 46)

Variables	Hospital *n* (%)	Primary health centre *n* (%)	Total *N* (%)
Age group (years) (mean ± standard deviation= 32 ± 6.43 years)			
18–25	3 (6.5%)	4 (8.7%)	7 (15.2%)
26–30	5 (10.9%)	10 (21.8%)	15 (32.7%)
31–35	7 (15.2%)	7 (15.2%)	14 (30.4%)
36–40	2 (4.3%)	2 (4.3%)	4 (8.6%)
>40	0	6 (13.1%)	6 (13.1%)
Marital status			
Married	11 (24%)	23 (50%)	34 (74%)
Unmarried	6 (13%)	6 (13%)	12 (26%)
Type of health care providers			
ANM/Senior ANM	4 (8.7%)	20 (43.4%)	24 (52.1%)
Staff nurse	12 (26.1%)	8 (17.4%)	20 (43.5%)
Psychosocial counsellor	1 (2.2%)	1 (2.2%)	2 (4.4%)
Participants educational qualification			
Proficiency certificate level in nursing	9 (19.5%)	13 (28.3%)	22 (47.8%)
ANMs	1 (2.2%)	6 (13%)	7 (15.2%)
High school (+2)	1 (2.2%)	1 (2.2%)	2 (4.4%)
Bachelor’s	5 (10.9%)	8 (17.3%)	13 (28.2%)
Master’s	1 (2.2%)	1 (2.2%)	2 (4.4%)
Number of patients attended per week by health care providers Median (Inter quartile range = 70) (IQR = 68.75)			
<20	7 (15.2%)	2 (4.4%)	9 (19.6%)
20–39	3 (6.5%)	3 (6.5%)	6 (13%)
40–59	1 (2.2%)	5 (10.8%)	6 (13%)
60–79	2 (4.3%)	5 (10.9%)	7 (15.2%)
80–99	0	1 (2.2%)	1 (2.2%)
≥100	4 (8.7%)	13 (28.3%)	17 (37%)
Working years in current health facility (years) (mean ± standard deviation= 4.94 ± 4.68 years)			
<1	1 (2.2%)	4 (8.7%)	5 (10.9%)
1–5	11 (23.9%)	16 (34.8%)	27 (58.7%)
>5	5 (10.8%)	9 (19.6%)	14 (30.4%)

### Health facility readiness-related findings

The results regarding health facility readiness are presented according to the health system building blocks, which have been adapted for this study.

#### Information

The average number of women who visited a public health facility for any kind of health service in the past month was 680 (Table 4). Three-fourth of health facilities reported IPV/DV cases in the past month. In a sample of 17 selected primary health care centres, 12 (70.6%) reported incidents of IPV or DV in the past month. Nine out of 11 public hospitals had separate recording and reporting systems for IPV while none of the primary health care centres had such systems, and the number of IPV cases was estimated by the providers. The majority of IPV/DV cases in health facilities were identified through victims’ self-disclosure, 64% while providing maternal health services and counselling. About 50% of the IPV/DV incidents reported to the providers were referred by the police (Table 4).

**Table 4.** Violence case identification-related information

Variables	Hospital *n* (%)	Primary Health Centre *n* (%)	Total *N* (%)
Number of women visiting the health facility in the past month (mean ± standard deviation= 680± 504.72)			
IPV/DV cases reported in past month			
Yes	9 (32.1%)	12 (42.9%)	21 (75%)
No	2 (7.1%)	5 (17.9%)	7 (25%)
Recording and reporting system for IPV/DV case			
Available	9 (32.1%)	0	9 (32.1%)
Not available	2 (7.2%)	17 (60.7%)	19 (62.9%)
Identification of IPV/DV case (mechanism of case identification)^a^			
Referral by Police	10 (35.8%)	4 (14.2%)	14 (50%)
Self-disclosure	9 (32.1%)	10 (35.7%)	19 (67.9%)
Identify while providing other service	5 (17.8%)	13(46.4%)	18 (64.2%)
Others^b^	3 (10.7%)	2 (7.1%)	5 (17.8%)

a Multiple responses.

b Other methods of identification of DV/IPV cases refers to physical examination while obtaining patient history or providing medical abortion and family planning services and also reports from family and community psychosocial workers.

### Service delivery

OCMC service was available in eight hospitals, providing counselling, medical care, drugs, lab and rehabilitation service such food, clothing and accommodation for women experiencing violence. Four of the OCMC facilities also covered transportation costs for survivors and six could refer victims to the police, three provided referrals to lawyers and two offered referral services to safe houses/shelters, if needed. Additionally, two of the OCMC sites provided dignity kits (towels, clothes, sanitary pads, combs, toothbrushes, toothpaste, underwear and soap) and coordinated care with the safe houses (Table 5). Regarding the primary health care centres, only two health care providers had received training in IPV management; however, they were inactive in the case management due to resource constraints. Consequently, when identifying women experiencing violence at these centres, counselling was provided based on the knowledge and education available, as the majority of health care providers in primary health care centres lacked training in violence management and psychosocial counselling.

**Table 5.** Health service delivery related to GBV

Variables	Hospital *n* (%)	Primary health centre *n* (%)	Total *N* (%)
Health facility with OCMC services (*n* = 28)			
Yes	8 (28.57%)	0	8 (28.57%)
No	3 (10.71%)	17 (60.71%)	20 (71.42%)
Services provided via OCMC centres (*n* = 8)			
Counselling	8	0	8
Medical care	8	0	8
Lab investigations	8	0	8
Rehabilitation service	8	0	8
Referral to police	6	0	6
Transportation cost	4	0	4
Referral to lawyer	3	0	3
Coordination with safe home	2	0	2
Dignity kit	2	0	2

#### Health workforce

Just below one-third (28%) of the providers were trained on management of GBV, more so at hospital compared to lower-level facilities, but nobody was trained specifically on the management of mental health consequences of IPV. Similarly, 17% of health care providers, 13% in hospitals and 4% in primary health care centres had psychosocial counselling skills. Given the limited mental health workforce in the country, psychiatrists or psychologists were only available in two (one large federal hospital and one provincial hospital) out of 28 health facilities, and only four hospitals and two primary health care centres had specific psychosocial counsellors (Table 6).

**Table 6.** Health workforce related to GBV and mental health problems management

Variables	Hospital *n* (%)	Primary health centre *n* (%)	Total *N* (%)
Participants trained in GBV (*N* = 46)			
Trained	9 (19.5%)	4 (8.7%)	13 (28.2%)
Untrained	8 (17.4%)	25 (54.3%)	33 (71.7%)
Participants trained in psychosocial counselling (*N* = 46)			
Trained	6 (13%)	2 (4.3%)	8 (17.3%)
Untrained	11 (24%)	27 (58.7%)	38 (82.7%)
Availability of psychiatrist or psychologist (*N* = 28)			
Available	2 (7.1%)	0	2 (7.1%)
Not available	9 (32.1%)	17 (60.7%)	26 (92.8%)
Availability of psychosocial counsellor (*N* = 28)			
Available	4 (14.2%)	2 (7.1%)	6 (21.4%)
Not available	7 (25%)	15 (53.5%)	22 (78.5%)
Perceived knowledge of participants about mental health (*N* = 46)			
Quite a bit	0	1 (2.1%)	1 (2.1%)
A fair amount	0	1 (2.1%)	1 (2.1%)
A moderate amount	1 (2.1%)	19 (41.3%)	20 (43.4%)
A little	8 (17.3%)	1 (2.1%)	9 (19.5%)
Very little	7 (15.2%)	5 (10.8%)	12 (26.0%)
Nothing	1 (2.1%)	2 (4.3%)	3 (6.5%)
Participants who have heard about OCMC (*N* = 46)			
Yes	13 (28.2%)	9 (19.5%)	22 (47.8%)
No	4 (8.6%)	20 (43.4%)	24 (52.1%)

**Figure 1.**
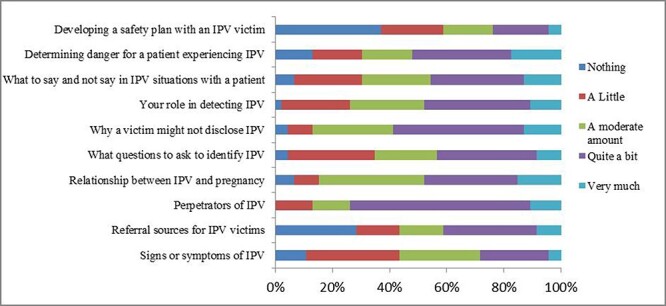
Perceived knowledge of health care providers in relation to IPV/DV management

More than half of the providers (52.1%) had not heard about OCMC. Approximately 70% of health care providers working in primary health care centres were not aware of the OCMC. Additionally, only a minority of participants demonstrated adequate knowledge of mental health (4.2%). Similarly, a limited percentage of participants (ranging from 4.34% to 15.21%) were familiar with developing safety plans for women experiencing violence, including understanding its relationship with pregnancy and recognizing signs and symptoms of IPV/DV (Table 6, Figure 1). Around two-thirds had substantial knowledge about the perpetrators of IPV/DV, while less than half (45.65%) were aware of the stigma and fear hindering women from disclosing IPV/DV. Moreover, more than one-fourth were unaware of anywhere to refer women who are experiencing violence.

**Figure 2.**
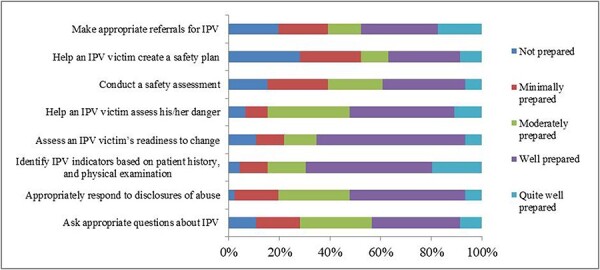
Perceived preparedness of health care providers to manage IPV/DV

Approximately half of the providers stated that they were well-prepared to respond appropriately to disclosures of abuse. Over two-thirds (69.56%) reported being prepared to identify indicators of IPV based on patient history and physical examination (Figure 2), but more than a quarter of health care providers (28.26%) felt entirely unprepared to assist victims in creating a safety plan.

*Human resources for psychosocial counselling to women experiencing violence*

The qualitative findings revealed that while only a few health facilities believed they had adequate staff to provide IPV services, many expressed readiness to deliver these services regardless, emphasizing the importance and impact of counselling for survivors of violence.

*Women, as members of society, endure various forms of violence, necessitating psychosocial counselling. Although I haven’t received training in violence management and psychosocial counselling, I’m keen on counselling women experiencing violence*. (Nursing inspector, primary health care centre, Parsa).

*Women experiencing violence undergo immense challenges, yet many refrain from disclosing their experiences of violence. Often, pregnant women visit us with explanations of accidents, but upon further exploration, they reveal abuse by their partners or in-laws. I am motivated to provide psychosocial counselling to these women. We already counsel women on family planning, so extending this to include psychosocial support for survivors of violence aligns with our duties*. (ANM, Primary health care centre, Sarlahi).

While some acknowledged great challenges to cope given the high number of patients seeking care and very limited staff.

*…I have to perform other duties too. Screening and finding cases of violence from the field and providing counselling to them requires a large amount of time, which as a nurse will be difficult. To make any gender-based violence-related program successful, a psychosocial counsellor is very important, especially in public hospitals* (Staff nurse, Hospital, Mahottari).

Health care providers working at OCMC also pointed out on availability of limited staff in the OCMC which makes their task difficult.

*As per the OCMC guideline there is sanctioned post of 3 staff nurses. In many OCMCs there is separate case manager, separate psychosocial counsellor from different NGOs like CMC Nepal, but here since the operation of OCMC, I have been working as a case manager, I also work in the coordination section, and I provide counselling as well. So, when I have to do all these, it is little hard for me to manage time (*Staff nurse, Hospital, Saptari).

*Sometimes there are 2–3 cases per day, and it gets little hectic on me. At times, it gets late. I have to stay here till night. And one thing is most of the cases of violence comes at night but I have to do it as I am the only staff in this section* (Staff nurse, Hospital, Rautahat).

Many providers had a positive attitude towards managing time to counsel women exposed to violence despite a high patient flow and hectic work schedule, except when the workload was particularly high.

*Almost every patient coming to access services is counselled in our facility; it comes under our job responsibility. Therefore, we can surely manage our time; it will not be very difficult for us to provide counselling to women experiencing violence* (ANM, Primary health care centre, Saptari).

*It’s crucial to offer psychosocial counselling to women experiencing stress, as without proper support, their condition can escalate to a critical stage, even leading to severe consequences such as death. Given appropriate training, I am motivated and interested in providing counselling to women facing violence*(Nurse, Primary health care centre, Bara).

#### Motivation of health workers in delivering psychosocial counselling: findings from qualitative interviews

In-depth interviews aimed at understanding the motivation of health care providers in delivering psychosocial counselling to women experiencing violence revealed several prominent themes.

### Prevalence of violence

Health care providers acknowledged the high prevalence of VAW in Madhesh Province. A majority of women in this province endure various forms of violence during their lifetimes. Particularly in marginalized communities, women are at increased risk due to deprivation of fundamental rights such as education, proper nutrition, health care and employment. This vulnerability exacerbates their susceptibility to violence and discrimination, leading to mental health issues.

### Sociocultural factors

Factors such as the low societal status of women, preference for male children, religious influences, particularly with the Muslim community and the prevalence of dowry were identified as major contributors to the high prevalence of VAW in Madhesh Province. These sociocultural aspects significantly impact the prevalence and perpetuation of VAW in this region.

*…women are often subject to male dominance and various forms of violence. On one occasion, a woman sought family planning (FP) counseling… However, she later returned and declined FP services because she had been physically abused by her husband due to seeking FP counseling* (Staff nurse, Primary health care centre, Rautahat).

*Many pregnant women attending antenatal care visits indirectly communicate their domestic issues and challenges to us. They articulate how their husbands and in-laws subject them to heavy workloads, provide inadequate nutrition, offer poor maternal care, and exacerbate the situation if the expected child is female* (ANM, Primary health care centre, Parsa).

A health care provider further expressed how women lie about their experience of violence. It is only after probing and developing trust, they tend to share about the abuse.

*Numerous women refrain from disclosing their encounters with violence. They tend to open up and share their experiences only when a sense of closeness or trust has been established. In numerous instances involving pregnant women seeking care, initial reports often mention accidental falls or mishaps. However, upon further exploration, these women reveal instances of abuse perpetrated by their partners or in-laws* (ANM, Primary health care centre, Sarlahi).

### Necessity of psychosocial counselling

Nearly all health care providers emphasized the critical role of psychosocial counselling in protecting women, mitigating violence and empowering women to confront violence. They highlight the emotional turmoil experienced by survivors of violence, including feelings of loneliness, extreme fear, mental stress, depression and suicidal ideation. Furthermore, providers unanimously agreed on the therapeutic benefits of appropriate counselling for violence survivors, aiding in their coping mechanisms and recovery. They stressed the significance of a safe environment for women to share their experiences and highlighted counselling’s role in raising awareness about women’s rights and combating the social acceptance of violence.

*Women typically refrain from discussing the violence they endure, often attempting to conceal it, leading to substantial anxiety and mental stress. Offering counseling would provide them a safe space to articulate their experiences of violence* (ANM, Primary health care centre, Bara).

*Many pregnant women attribute their falls to accidents initially, but upon detailed inquiry, they disclose instances of physical violence inflicted by their husbands and in-laws. Hence, counseling could aid in investigating such occurrences and guiding them to respond appropriately in such situations* (ANM, primary health care centre, Sarlahi).

### Motivation for counselling women

Many health workers were driven to offer opportunities for women, empowering them to counteract violence and aiding them in achieving enhanced health and well-being. These motivations are not solely driven by a sense of duty but are deeply intertwined with firsthand observations of the transformative effects of counselling. Providers express a collective aspiration to contribute meaningfully to women’s welfare, acknowledging the province’s existing shortcomings and recognizing counselling as a pivotal tool to redress these issues.

*Our province lags behind others in various aspects, and unfortunately, violence is widespread. This counseling could offer us the chance to work towards women’s welfare and contribute to improving their well-being* (Staff Nurse, Primary health care centre, Saptari).

*I am highly motivated to provide counseling as it will undoubtedly assist in rescuing and safeguarding women from violence and its repercussions* (ANM, Primary health care centre, Sarlahi).

One health provider shared feeling motivated after observing changes in patients who underwent counselling, further inspiring her to conduct more counselling sessions.

*I’ve witnessed cases where women experiencing violence have transformed after attending counseling sessions. When we witness such positive changes in women’s lives, it serves as additional motivation for us* (Staff Nurse, Hospital, Dhanusha).

#### Medical products, vaccines and technologies

More than half of the public health facilities (64%) had separate rooms for physical and genital examinations and 46% had separate rooms for counselling (Table 7). Most hospitals, and thereby a majority of the OCMC sites (seven out of 11), allocated separate rooms for physical and genito-anal examinations, along with counselling spaces dedicated to managing IPV cases. In contrast, primary health care centres lacked dedicated counselling rooms, as the OCMC service was not available at these centres. It was anticipated that, considering the provision of family planning services in all public health facilities across the country, separate counselling rooms would be available.

**Table 7.** Available resources for violence and mental health case management in health facilities (*n* = 28)

Variables	Hospital *n* (%)	Primary health centre *n* (%)	Total *N* (%)
Separate room for physical examination			
Yes	7 (25%)	11 (39.3%)	18 (64.3%)
No	4 (14.3%)	6 (21.4%)	10 (35.7%)
Designated room for genito-anal examination			
Yes	7 (25%)	12 (42.9%)	19 (67.9%)
No	4 (14.3%)	5 (17.8%)	9 (32.1%)
Separate room for counselling			
Yes	7 (25%)	6 (21.4%)	13 (46.4%)
No	4 (14.3%)	11 (39.3%)	15 (53.6%)
Availability of information, education and communication materials			
Yes	8 (28.6%)	1 (3.6%)	9 (32.2%)
No	3 (10.7%)	16 (57.1%)	19 (67.8%)
Emergency contraception			
Yes	9 (32.1%)	1 (3.6%)	10 (35.7%)
No	2 (7.2%)	16 (57.1%)	18 (64.3%)
Tetanus prophylaxis			
Yes	9 (32.1%)	6 (21.5%)	15 (53.6%)
No	2 (7.2%)	11 (39.2%)	13 (46.4%)
Hepatitis B			
Yes	3 (10.8%)	0	3 (10.8%)
No	8 (28.6%)	17 (60.7%)	25 (89.2%)
Screening for STIs	11 (39.2%)	17 (60.8%)	28 (100%)
Screening for HIV	11 (39.2%)	17 (60.8%)	28 (100%)
Mental health-related drugs			
Available	3 (10.7%)	2 (7.1%)	5 (17.8%)
Not available	8 (28.6%)	15 (53.6%)	23 (82.2%)
Continuous supply of electricity			
Yes	11 (39.2%)	15 (53.6%)	26 (92.8%)
No	0	2 (7.2%)	2 (7.2%)
Availability of guidelines for IPV/DV case management			
Yes	5 (17.8%)	0	5 (17.8%)
No	6 (21.4%)	17 (60.7%)	23 (82.1%)
Availability of guidelines for mental health case management			
Yes	2 (7.2%)	0	2 (7.2%)
No	9 (32.1%)	17 (60.7%)	26 (92.8%)

The national clinical protocol on GBV (Ministry of Health and Population, 2015) recommends the provision of essential medical services such as emergency contraception and tetanus prophylaxis to be universally available at all health facilities regardless of level. However, this assessment revealed a scarcity of crucial medical tools for women exposed to violence, with low supplies of emergency contraception (36%), tetanus prophylaxis (54%) and hepatitis B vaccine (11%). While over 80% of hospitals provided emergency contraception, only one primary health care centre offered the same service. Tetanus prophylaxis was available in all but two hospitals and in just over one-third of primary health care centres. Nevertheless, all public health facilities could conduct screening for sexually transmitted infections, hepatitis B virus and HIV (Table 7). Despite the inclusion of psychotropic medicines such as antipsychotics, antidepressants, anxiolytics, mood stabilizers and antiepileptic drugs in the essential medicines list (World Health Organization, 2022b), only 18% of the health facilities assessed had these medications available for treating mental health conditions.

#### Leadership and governance

Educational materials and standard guidelines related to GBV case management were lacking in all primary health care centres and in most hospitals (6 out of 11) (Table 7). A clinical protocol on GBV has been developed in the country, intended for adoption by health facilities at all levels. However, none of the primary health care centres possessed guidelines for GBV management or informational, educational and communication (IEC) materials for counselling. However, health care providers reported that access to such training materials, guidelines and suitable environments for delivering GBV counselling services would increase their interest and encourage them to provide more effective counselling. Only 7% of the selected public hospitals and primary health care centres reported having guidelines available for mental health care management.

*I find counselling very interesting as I have been doing it in my regular duty. I counsel regarding antenatal care, family planning and many other aspects. It would further my abilities if we get training for delivering psychosocial counselling to women experiencing violence. If guidelines and relevant materials are also provided to us, I think it would be even more effective* (Staff Nurse, Primary health care centre, Sarlahi).

Most expressed interest in offering psychosocial counselling to clients exposed to violence and some providers had worked in health service delivery for a very long time and counselled patients as a regular duty.

*As I have been working here for many years, I am quite familiar with local people and their issues. Additionally, women residing in this area trust me and listen to my suggestions. Therefore, I am interested to provide such counselling to violence survivors* (ANM, Primary health care centre, Dhanusha).

*Counselling could provide us with the opportunity to listen and learn from the patient’s experiences and suffering. Therefore, I find it very interesting* (Staff Nurse, Hospital, Dhanusha).

#### Coordination

**Figure 3.**
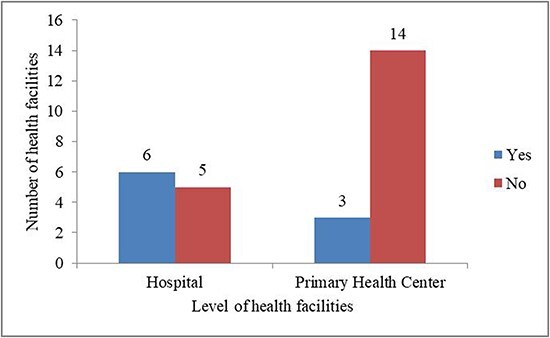
Provision for referring women experiencing violence to psychiatrists or psychologists (*n* = 28)

Only 32% of health facilities (Figure 3) referred women exposed to violence to another facility, e.g. a provincial or large hospital for medical treatment and psychosocial help when needed.

*We serve as a referral mechanism to higher-level hospitals or authorities for violence and mental health management services, although our training for awareness about available services at higher levels remains limited (*Staff nurse, Hospital, Siraha).

*The issue lies in the inadequate dissemination of information about available services within our hospital and to other health facilities (primary health care centres, health post, urban health clinic). This obstructs smooth referral mechanisms between hospital departments and public health facilities* (Staff Nurse, Hospital, Mahottari).

Qualitative interviews revealed that health care providers working at OCMC were knowledgeable about referral sources. They mentioned referring cases to the police, lawyers and safe houses as needed. In contrast, health care providers at primary health care centres demonstrated less awareness. While some indicated a willingness to refer violence cases to the police, others admitted a lack of knowledge about referral points for women exposed to violence.

Health care providers at OCMC in various hospitals shared their experiences:

*Given my work in violence against women and mental health as a psychosocial counsellor, I am familiar with referral locations. I have collaborated with the police, safe houses, and other organizations dedicated to combating violence against women* (Psychosocial Counsellor, Hospital, Sarlahi).

*When violence cases come to me, I follow the OCMC guidelines for treatment and counselling. Subsequently, I refer cases to the police, government lawyers, and sometimes to safe houses (Manav Sewa Ashram)* (Staff Nurse, Hospital, Rautahat).

In contrast, health care providers at primary health care centres expressed different views:

*Women are enduring significant violence. Some cases involve poison consumption and even suicide attempts. Due to resource limitations here, I refer such violence cases to OCMC sites* (Staff Nurse, Primary health care centre, Mahottari).

*We haven’t received any training related to violence against women here, so I’m uncertain about where to refer women facing violence* (Staff Nurse, Primary health care centre, Rautahat).

*For cases of physical assault, we refer and report them to the police. However, we’ve noticed women seeking help within women’s groups before approaching us, and from here, we direct the cases to the police* (ANM, Primary health care centre, Sarlahi).

## Discussion

To the best of our knowledge, this is the first study conducted in Nepal to evaluate the readiness of public health facilities in identifying and managing GBV, as well as addressing the associated mental health consequences. Additionally, the study explored the motivation of health care providers in providing psychosocial counselling to women impacted by violence. This study’s findings revealed that only 28% of the public health facilities assessed were equipped to deal with cases of violence and these were primarily hospitals with OCMC, which provided a range of services including counselling, medical care, lab investigations, rehabilitation and referral services. In contrast, health facilities without OCMC were found to be lacking in these services, with a limited capacity to manage, refer, record and report cases of violence. Despite the scarcity of resources in public hospitals and primary health care centres in Madhesh Province for handing VAW, health care providers are willing to offer such services, including psychosocial counselling, provided they receive appropriate training and resources.

In Nepal, an OCMC operational manual was developed in 2011 by the Ministry of Health and Population (Ministry of Health and Population and Population Division, 2011), and a national clinical protocol was established in 2015 (Ministry of Health and Population, 2015). However, many health facilities did not have access to these guidelines and protocols, which made it challenging to manage GBV cases comprehensively. These findings were similar to those of a study conducted in Palestine (Colombini *et al*., 2020).

For any intervention to be effectively implemented there must be an adequate number of willing and capable staff. However, in three-quarters of the health facilities assessed, trained human resources to manage GBV were absent which is a significant barrier to service provision (Catak Taskiran *et al*., 2019). Additionally, in some primary health care-level facilities, limited staffing prevented health care providers from having the time to ask questions and provide counselling to patients. While counselling may be one way of identifying GBV victims, the lack of time due to limited staff or high patient load presents a challenge to effective counselling (Briones-Vozmediano *et al*., 2021; Anguzu *et al*., 2022). The health facilities providing OCMC services noted the challenges of limited staffing within the OCMC, which hinders their work. On the positive note, certain health care providers expressed readiness to incorporate psychosocial counselling into their routine responsibilities, provided they receive training in managing violence. Additionally, some health care providers acknowledged that aiding women facing violence is an inherent part of their responsibility. Public hospitals equipped with OCMCs demonstrated readiness in managing cases of violence. Conversely, primary health care centres exhibited a lack of preparedness for both violence management and referrals. Despite the development of a clinical protocol for managing GBV intended for implementation across all health facility levels, its current implementation rate is notably low. Moreover, primary health care centres not only face challenges in managing all forms of violence but also lack basic knowledge regarding the identification of female survivors of violence, conducting screenings, providing emergency contraception, tetanus prophylaxis and offering referral services for additional care and support.

The study found that many health care providers had limited knowledge about how to screen and treat women experiencing violence, which is consistent with studies conducted in Uganda, India and Tanzania (Kaye *et al*., 2005; Sharma *et al*., 2018; Ambikile *et al*., 2020). Additionally, a similar study conducted among medical doctors in Nigeria demonstrated low knowledge and preparedness on all items included in PREMIS (Omodele *et al*., 2015). A study conducted among women receiving routine antenatal care in Nepal revealed that <20% of participants were questioned about violence by health care providers (Rishal *et al*., 2018). This finding indicates a deficiency in the knowledge and preparedness of health care providers to address cases of VAW.

The availability of adequate infrastructure is equally important for the proper service delivery and management of GBV cases (Deosthali-Bhate *et al*., 2018). However, the majority of public health facilities do not have dedicated rooms for physical examination, genito-anal examination and counselling, especially for GBV management, and often share the use of antenatal care and labour rooms for cases of GBV. Such a lack of space has also been noted as a challenge to GBV management in other countries (Colombini *et al*., 2020; Anguzu *et al*., 2022). Additionally, IEC materials were unavailable in ∼70% of health facilities assessed, although they are considered essential for effective counselling and increasing knowledge and awareness among health staff (Hewitt, 2015). IEC materials have been identified as an effective method to inform women experiencing violence about available services (Yaker, 2019). These materials have been prominently displayed on the health facility walls, guiding women on whom to approach regarding their experiences of violence (World Health Organization, CEHAT and HRP, 2021). Moreover, they serve to emphasize the availability of assistance for women experiencing violence, affirming their right to a violence free life (Gadappa *et al*., 2022). In Nepal, similar IEC materials have been employed to disseminate information about OCMC and their associated services (Ministry of Health and Population, 2017).

It is recommended by the national clinical protocol on GBV that women experiencing violence visiting health facilities must be offered emergency contraception, tetanus prophylaxis, hepatitis B vaccine and be screened and treated for different sexually transmitted infections, including HIV (Ministry of Health and Population, 2015). Our findings revealed significant gaps in service availability. For instance, emergency contraception was accessible in only one-third of the health facilities, predominantly hospitals. Tetanus prophylaxis was offered in approximately half of the facilities, with over 80% of hospitals providing this service compared to one-third of primary health care centres. Additionally, hepatitis B vaccination was available. A similar gap was reported in the World Bank report based on studies from South Asia, including Nepal (Solotaroff and Pande, 2014). On a positive note, many of the public health facilities were equipped with labs and could thus screen for major sexually transmitted infections such as syphilis, HIV and hepatitis B virus.

There is also a need to improve coordination among related actors involved in GBV case management. Very few public health facilities work in close collaboration with the police, lawyers or safe houses, similarly observed in India (Deosthali-Bhate *et al*., 2018) and a report across South Asia (Solotaroff and Pande, 2014).

Many health care providers in our assessment were interested and motivated in delivering psychosocial counselling to women experiencing violence, primarily listing the possibility to identify victims of violence, support and empathize with these women and boost their self-confidence. By providing psychosocial counselling they hope to contribute to women’s well-being, help them solve their problems and protect them from more violence. A study in India also reported similar motivating factors, highlighting the importance of trust between providers and clients (Gadappa *et al*., 2022).

A multi-country study highlighted the varied responses of health system to VAW across different countries, each with its unique approach. However, universally, enhancing the health system was recognized as imperative (García-Moreno *et al*., 2015). The study underscored that the presence of protocol within health facilities for managing VAW, capacity building for health care providers and the establishment of effective coordination among referral networks and agencies were pivotal components in addressing this issue. Our current study revealed a deficiency in guidelines for guideline for GBV management in numerous health facilities. Primary health care providers and hospitals lacking OCMCs showed limited awareness in handling cases of violence and providing psychosocial counselling. Moreover, the referral networks exhibited weakness and uncertainty in identifying appropriate channels for cases of violence and individuals with mental health issues.

Similarly, a study assessing the readiness of health care providers in addressing VAW indicated that readiness involves a provider’s commitment to addressing violence, adoption of an advocacy approach, trustworthiness, ability to collaborate within a team and full support from the health system (Hegarty *et al*., 2020). In our study, health care providers displayed motivation and interest in managing violence and delivering psychosocial counselling, yet they faced challenges due to an unsupportive health system. Many health facilities lacked essential resources, including adequate human resources, hindering their ability to address these issues effectively.

The readiness of health care providers to address violence relied on various factors such as self-efficacy, emotional readiness, motivation and attitudinal readiness (Leung *et al*., 2018). Conversely, preparedness encompasses their knowledge and communication skills. In our study, health care providers exhibited limited knowledge in managing violence. Many lacked expertise in developing safety plans and establishing referral networks. However, they expressed motivation to deliver psychosocial counselling and other services for addressing VAW if assured of the availability of necessary resources in line with the GBV guideline of the country.

Assessment of health facility readiness was conducted using WHO’s health system building blocks framework. Findings revealed that particularly primary health care centres were not prepared to respond to VAW. They lacked trained personnel, essential infrastructures such as separate counselling rooms, basic mental health-related medications and emergency contraception services, despite GBV management guidelines recommending the provision of these services even at primary health care centres. Moreover, their knowledge regarding referral sources was limited, and necessary forms and registers for recording and reporting violence cases were unavailable. Similar inadequacies were observed in hospitals without OCMCs. Conversely, hospitals with OCMC-managed violence cases were understaffed but offered staff training in violence management, provided of psychosocial counselling in separate rooms, and established referral networks. Furthermore, they maintained necessary documentation through forms and registers for recording violence-related information.

For future assessments, employing an interpretive policy analysis approach (Yanow, 2000) in the readiness analysis of GBV management programs could be beneficial for gaining deeper insights into the gaps between policy practices. This approach would aid in evaluating whether health facilities possess the resources, logistics, health workforces and referral sources as recommended in the country’s clinical protocol or guidelines for GBV management. Ensuring that health facilities possess these resources could significantly contribute to strengthening the health system. Conversely, if health facilities lack these resources as per the guidelines or protocol, exploring implementation challenges and reasons behind non-implementation would be crucial.

## Strengths and limitations

This study has been helpful in assessing the readiness status of the selected public health facilities to identify and manage cases of GBV, and it could guide future interventions for GBV management at health systems level. Furthermore, it could be useful for policymakers in developing responses for GBV management. However, the study had some limitations. First, we could not assess all the public health facilities of Madhesh Province, so the generalizability of our findings is uncertain, but facilities in both rural and urban areas and all public hospitals except one were included, covering the majority of the health facilities in the province. Second, although it was envisaged to interview two health care providers from each public health facility, we could not reach them all since they were selected based on purposive sampling, and only those who were present on the day of the health facility visit were included in the assessment.

In summary, our study revealed that the majority of public health facilities in this high GBV burden Nepalese province were inadequately prepared to identify, manage and refer victims of violence. However, the high motivation among health care providers to offer psychosocial counselling to women affected by GBV is undermined by the lack of policy-to-practice implementation, exemplified by insufficient resources needed to execute services outlined in the country’s clinical protocol on GBV. This underscores the urgent need for targeted interventions aimed at training and guiding health care providers in addressing VAW. Concurrently, there is a crucial necessity to fortify health facilities in managing GBV by ensuring the availability of well-trained human resource within a supportive health system. This includes the development and provision of essential infrastructures such as dedicated counselling rooms, access to IEC materials and the establishment of comprehensive registers and forms for documenting women’s experiences of violence and the care they receive.

Moreover, primary health care centres can serve as a vital intermediary between higher-level hospitals and women experiencing violence. They can facilitate referrals for advanced medical treatment related to mental health issues. Additionally, these facilities can play a pivotal role in linking women with safe shelters, local law enforcement agencies, legal counsel and judicial bodies for comprehensive management of violence.

## Data Availability

The data underlying this article will be shared on reasonable request to the corresponding author.

## Abbreviations

GBV: Gender-Based Violence

IPV: Intimate Partner Violence

DV: Domestic Violence

WHO: World Health Organization

PREMIS: Physician’s Readiness to Manage Intimate Partner Violence Survey

OCMC: One-stop Crisis Management Centre

VAW: Violence against Women

ANM: Auxiliary Nursing Midwives

HIV: Human Immunodeficiency Virus

## Reflexivity Statement

As co-authors of this article, we acknowledge our own professional backgrounds and personal experiences which may have influenced our research process, interpretation of findings and writing. Dr Keshab Deuba, the lead author, has an MMSc in Epidemiology and a PhD in Public Health. Dr Deuba’s work focuses on health policy and systems research, particularly enhancing surveillance and evaluation of interventions targeting communicable diseases, including HIV and tuberculosis, and addressing key global health challenges like mental health and gender-based violence in low- and middle-income countries. Dr Deuba brings over 10 years of experience from various roles in international development organizations, universities and UN agencies. R.S., R.K., A.L., B.K.J. and D.M. have extensive experience in public health, with a focus on the prevention and management of GBV and mental health problems among vulnerable and marginalized populations in low- and middle-income countries. A.M.E. is a professor from Sweden with experience working collaboratively with public health experts from diverse regions. Majority of the co-authors for this study are female, except K.D., A.L. and B.K.J. and have a commitment to promoting gender equity in health research and practice. We strived to maintain reflexivity throughout the study by regularly discussing our own biases, assumptions and potential power imbalances during data collection, analysis and interpretation. We also sought feedback from external reviewers to enhance the rigor and credibility of our work.
